# A Nearly Missed Case of Mitral Bioprosthetic Paravalvular Leak (PVL)-Related Waring Blender Syndrome Treated With Transcatheter Mitral PVL Closure

**DOI:** 10.7759/cureus.57552

**Published:** 2024-04-03

**Authors:** Sowjanya Kalluri, Hamid S Shaaban, Addi Suleiman, Samer Jumean, Gunwant Guron

**Affiliations:** 1 Hematology and Oncology, Saint Michael's Medical Center, Newark, USA; 2 Cardiology, Saint Michael's Medical Center, Newark, USA; 3 Internal Medicine, Saint Michael's Medical Center, Newark, USA; 4 Internal Medicine, New York Medical College, Valhalla, USA

**Keywords:** transcatheter mitral valve closure, waring blender syndrome, intravascular hemolysis, prosthetic valve, hemolytic anemia

## Abstract

A 72-year-old woman with recently diagnosed non-small cell lung cancer, who underwent cardiac bypass and bioprosthetic mitral valve replacement presented to our cancer center with lightheadedness, severe fatigue, and shortness of breath. Initial blood tests showed mild hemolytic anemia. The patient also complained of occasional bright red bleeding per rectum. Esophagogastroduodenoscopy and colonoscopy did not reveal an acute source of bleeding. An initial transesophageal echocardiogram did not show significant valvular or paravalvular abnormalities. Meanwhile, the patient's hemolytic anemia worsened. She received eight units of packed red blood cell transfusions. Schematic workup for hemolytic anemia revealed negative Coomb’s test, positive urine hemosiderin, normal ADAMTS13 activity, and absent splenomegaly. A relook of the patient's transesophageal echocardiogram (TEE) showed a small paravalvular leak of the bioprosthetic mitral valve. The patient was referred to a tertiary center, and repair of the perivalvular leak with glue resolved her hemolytic anemia, subsequently improving the lab values, symptoms, and quality of life. This case highlights the schematic workup of hemolytic anemia and also the importance of recognizing the association between hemolytic anemia and valvular abnormalities.

## Introduction

Waring blender syndrome refers to the destruction of red blood cells, which can become life-threatening. The syndrome often occurs in people with dysfunctional prosthetic heart valves and can cause non-autoimmune intravascular hemolytic anemia [1]. The red blood cells are hemolysed due to a dysfunctional prosthetic heart valve [1]. Hemolytic anemia is a known complication after mechanical valve replacement. However, the incidence of early onset of severe transfusion-dependent symptomatic hemolytic anemia is sporadic following a bioprosthetic mitral valve replacement. A paravalvular leak is a condition when the blood flows backward between the heart tissue and the prosthetic heart valve. The paravalvular leak is a rare complication related to the surgical replacement of mitral and aortic valves [2]. The incidence is around 5-15% of the cases. Most of the time, these leaks are not harmful to the patient. However, large leaks may lead to heart failure, while more minor leaks can lead to hemolytic anemia [2]. This case highlights the importance of being aware of the possibility of paravalvular leaks as a cause of hemolytic anemia, and timely diagnosis and treatment are crucial for the patient's well-being.

## Case presentation

We report a case of a 72-year-old woman who had a past medical history of smoking, asbestos exposure, hypertension, hypothyroidism, severe coronary artery disease with congestive heart failure, and severe mitral regurgitation. She initially came to our cancer center for the management of lung cancer. A recent biopsy of the lung nodules was performed, which showed adenocarcinoma of the lung, with positive thyroid transcription factor-1 (TTF-1) and programmed death-ligand 1 (PDL1) >5%. She was diagnosed with stage IV metastatic lung cancer. Before initiating chemoimmunotherapy, the patient underwent coronary artery bypass grafting and mitral valve replacement with a bioprosthetic valve. Postoperatively, the patient's hemoglobin dropped from a baseline of 10.5g/dL to 7.4 g/dL, supported by blood product transfusion.

Later, the patient came to the emergency room with hypotension, diaphoresis, and severe fatigue. The initial workup showed a decrease in hemoglobin from 10 g/dL to 7.1 g/dL. Markers of hemolysis were elevated, with lactate dehydrogenase (LDH) at 713 U/L, a haptoglobin less than 31 mg/dl, and a high reticulocyte count of 3.2%. The patient was started on oral iron and folate supplementation. Esophagogastroduodenoscopy (EGD) and colonoscopy revealed internal hemorrhoids with no active bleeding stigmata, which was not significant enough to explain the patient's severe anemia. A transesophageal echocardiogram (TEE) was performed due to concern for valve-associated hemolysis, which showed a well-positioned bioprosthetic valve with an area of 3.5 cm^2^ but with damaging paravalvular leaks or regurgitations.

**Figure 1:**
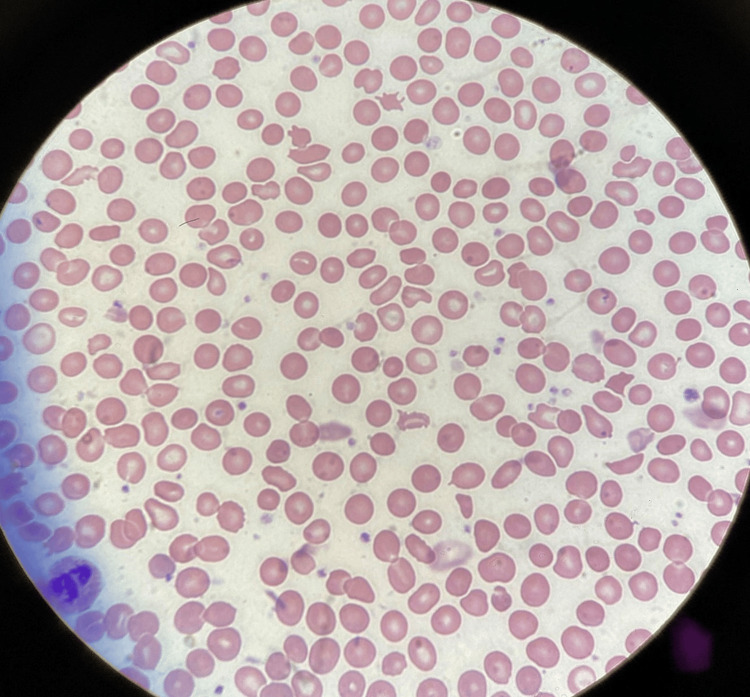
Peripheral blood smear of the patient obtained during hemolysis showing multiple schistocytes.

Meanwhile, the patient received one dose of chemoimmunotherapy with carboplatin/pemetrexed and pembrolizumab for lung cancer. A week later, she again presented to the cancer center with symptomatic anemia. Scleral icterus and left-sided holosystolic murmur were noted on exam. The patient was emergently sent to the ER and found to have a worsened hemolytic picture with a Hb of 6.9 g/dL, elevated LDH 1995 U/L, and low haptoglobin at <7.75 mg/dL with a reticulocyte count of 6 and total bilirubin elevated at 2.1 mg/dL. Peripheral blood smear showed multiple schistocytes (Figure 1). Direct Coombs test was negative for antibody. Urine hemosiderin was strongly positive, indicating intravascular hemolysis. The patient's ADAMTS13 level was normal at 72.2% in addition to normal platelet and white blood cell count. A CT of the abdomen showed a normal-sized spleen. The fibrinogen level was mildly elevated at 444 mg/dL. The patient required eight units of packed red blood cells (pRBCs) in only two months for severe hemolytic anemia despite being on iron and folate supplementation. All of these events occurred after the mitral valve replacement. Upon re-examination of the TEE, a small paravalvular leak was found between 10 and 11 o'clock on 3D images of the mitral valve. The patient was sent to a tertiary medical center, where the leak was confirmed, and PVL closure was performed using amplatzer vascular plug II (AVP‐II) 8 mm via right percutaneous femoral access. The patient's hemolytic anemia improved with improved hemoglobin, haptoglobin, and LDH levels. As early as five days after the procedure, the patient reported a significant improvement in her symptoms, with normalization of haptoglobin and reticulocyte count and LDH levels.

## Discussion

Our patient was diagnosed with a bioprosthetic mitral paravalvular leak as the cause of her early onset severe transfusion-dependent hemolytic anemia. Our case highlights the importance of a systematic approach to diagnosing hemolytic anemia in patients with multiple comorbidities, including severe coronary artery disease, mitral regurgitation, and metastatic lung cancer. It also emphasizes the need to review and reexamine the scans and images to prevent delays in treatment and reduce morbidity and mortality.

A paravalvular leak is a well-established cause of hemolytic anemia in patients with prosthetic heart valves, a condition commonly known to occur in the early postoperative period. Although mitral paravalvular leaks, for the most part, remain clinically insignificant, in some patients, they can cause life-threatening hemolysis and regurgitation-induced heart failure [3]. These complications arise due to increased shear stress on red blood cells, resulting from turbulent flow through the defect that causes mechanical trauma and red blood cell fragmentation [4]. These fragmented cells are seen as schistocytes on the peripheral blood smear. Clinically significant hemolysis is commonly seen in smaller defects that cause high-velocity jets. Patients with preexisting anemia due to nutritional deficiencies are at further risk due to increased red blood cell fragility. Anemia further increases the turbulence because of reduced blood viscosity and a compensatory increase in cardiac output. Mechanical damage to RBCs occurs because of blood-flow disturbances, rapid acceleration and deceleration of the regurgitant jet, and high shear stresses on the cell membrane. Because of these torrential blood currents, this condition is known as the Waring blender syndrome. [1]. A Coombs test is almost always negative. Elevations in urine hemosiderin occur due to intravascular hemolysis.

A pan systolic murmur is almost always considered abnormal in patients with prosthetic valves and is an indication for evaluation with echocardiography [5]. TEE is the initial test of choice for suspected paravalvular leaks [6]. The presence of a turbulent eccentric jet that originates outside the prosthetic sewing ring is the ideal representation of this condition. However, the shadowing of the sewing ring or annular calcification can cause a falsely normal depiction of the valve in a TEE. A TEE is often necessary to provide a definitive diagnosis. TEE further aids in assessing the function of the valve, evaluating the severity of regurgitation, and accurately localizing the defect [7]. Symptoms of heart failure, such as dyspnea and symptomatic hemolytic anemia, are two critical indications for percutaneous PVL closure [5]. The American College of Cardiology and the American Heart Association recommend percutaneous repair of paravalvular prosthetic valve regurgitation for patients with intractable hemolysis or heart failure symptoms NYHA class III/IV [6]. Such patients are at high risk for surgical intervention and possess anatomic features suitable for catheter-based therapy when performed in centers with expertise in this procedure [5]. In symptomatic patients, even a modest therapeutic reduction in regurgitation may be sufficient to restore them to their previous compensated state [8]. Percutaneous closure of symptomatic paravalvular leaks has demonstrated immediate and long-term success and is effective in managing symptoms of heart failure and hemolytic anemia [6].

Paravalvular leak and Waring blender syndrome are the surgical complications of cardiac valve replacements and usually occur rarely with bioprosthetic valves. Our patient presented with very severe anemia, requiring eight units of blood transfusion within two months after the valve replacement [7]. Statistically, paravalvular mitral leaks occur more frequently (7-17%) than paravalvular aortic leaks (2-10%) and are more common in mechanical valves than bioprosthetic valves. Clinicians should pay close attention to any new symptoms that may indicate the presence of these leaks. Surgical or percutaneous repair has been shown to significantly improve patient outcomes compared to medical treatment alone [8].

## Conclusions

Our case highlights the systematic approach to identifying the causes of hemolytic anemia. It is crucial to pay attention to the temporal relationships of hemolysis to any surgical procedure, cardiac valve replacement, or device insertion. This can help identify the cause and rectify it, resulting in marked clinical improvement along with laboratory findings, as exemplified in our case.
